# Analytical Validation of Low-Cost Optical Sensors for Freshwater Monitoring: A Scoping Review of Current Gaps and a Proposed Framework

**DOI:** 10.3390/s26123846

**Published:** 2026-06-17

**Authors:** Riccardo Gaetano Cirrone, Amedeo Boldrini, Alessio Polvani, Xinyu Liu, Francesco Vesprini, Luisa Galgani, Anna Witter, Óscar González, Gabriella Tamasi, Steven Arthur Loiselle

**Affiliations:** 1 Department of Earth and Marine Sciences, University of Palermo, Via Archirafi 22, 90123 Palermo, Italy; riccardogaetano.cirrone@unipa.it; 2 Department of Biotechnology, Chemistry and Pharmacy, University of Siena, Via Aldo Moro 2, 53100 Siena, Italy; alessio.polvani@student.unisi.it (A.P.); x.liu@student.unisi.it (X.L.); f.vesprini@student.unisi.it (F.V.); luisa.galgani@unisi.it (L.G.); gabriella.tamasi@unisi.it (G.T.); steven.loiselle@unisi.it (S.A.L.); 3 Center for Colloids and Surface Science (CSGI)-Siena Research Group, University of Florence, Via Della Lastruccia 3, 50019 Firenze, Italy; 4 Earthwatch Europe, 102–104 St. Aldate’s, Oxford OX1 1BT, UK; awitter@earthwatch.org.uk; 5 Fab Lab Barcelona, Carrer de Pujades 102, 08005 Barcelona, Spain; oscar@fablabbcn.org

**Keywords:** low-cost, IoT, water quality, open-source, analytical validation

## Abstract

Low-cost optical sensors have emerged as promising tools for in situ freshwater quality monitoring, offering the potential to expand spatial and temporal data coverage, particularly in community-based monitoring projects. However, despite rapid technological development of low-cost optical sensors, analytical validation practices of these devices remain poorly studied. This study aims to systematically and critically assess analytical validation practices applied to low-cost optical sensors based on absorbance, fluorescence, colorimetry, and light scattering, potentially designed for community-based freshwater monitoring. A total of 40 studies were analysed to evaluate how key analytical performance parameters, including sensitivity, accuracy, precision, and repeatability, as well as comparison with reference methods or benchtop instruments, were assessed and reported in relation to established validation guidelines. The analysis revealed substantial heterogeneity and critical gaps in validation approaches. While most studies report sensitivity metrics such as limits of detection and quantification, comprehensive evaluation of key analytical parameters such as accuracy, precision, and reproducibility was often limited. The reliance on single calibration experiments and high determination coefficients ($R^{2}$) frequently overestimates sensor performance. The lack of open-source materials further limits reproducibility and deployment: essential information such as design files, calibration procedures, and open-source resources is often incomplete or unavailable. To address these limitations, we propose a structured framework for validation and reporting that integrates established analytical guidelines with the practicalities of low-cost sensor development. Adoption of this approach would enable more consistent performance evaluation, improving reproducibility and facilitating comparison across studies and devices. Overall, strengthening analytical validation and reporting practices is essential to support the transition of low-cost optical sensors from proof-of-concept systems to reliable analytical devices for freshwater quality monitoring.

## 1. Introduction

Freshwater systems play a critical role in sustaining ecological processes and human activities, yet their quality is increasingly threatened by climate change and a range of anthropogenic pressures [1]. Effective monitoring of water quality is therefore essential, but conventional approaches are often limited by high costs, low spatial resolution, and restricted coverage, particularly in small or remote catchments [2]. In recent years, community-based monitoring has emerged as a complementary approach to conventional monitoring, enabling the collection of environmental data at improved spatial and temporal scales [3,4]. However, the reliability and comparability of data generated through community-based monitoring remain a central challenge [5]. Many existing initiatives rely on low-cost, user-friendly tools, such as colorimetric kits, which provide only semi-quantitative results and are subject to user-dependent variability [6,7]. To address these limitations, a growing body of research has focused on the development of low-cost optical sensors for in situ water quality monitoring [8,9]. These devices leverage accessible components, including light-emitting diodes, photodetectors, and smartphone-based detection systems, and are increasingly evaluated for their analytical performance in terms of sensitivity, often combined with open-source hardware and software platforms [10]. Previous reviews have primarily examined the technological aspects of these systems, including sensor design, cost reduction strategies, and integration with Internet of Things (IoT) architectures [11,12]. Despite rapid technological progress, significantly less attention has been given to the analytical validation practices of these sensors. In particular, there is limited understanding of whether low-cost optical sensing platforms meet established criteria for analytical performance, such as accuracy, precision, repeatability, and comparability with reference methods. Moreover, the extent to which validation procedures are reported in a transparent and reproducible manner remains unclear. This gap is critical, as insufficient or inconsistent validation can limit the practical applicability of these sensors, regardless of their technological innovation. Without robust validation, it is difficult to assess measurement reliability, compare results across studies, or support broader deployment in distributed monitoring networks. Unlike previous works, which focus primarily on hardware and application domains, the present study uses a scoping review approach since it systematically maps recent low-cost optical sensors for freshwater quality monitoring and evaluates how validation is performed and reported, with emphasis on methodological rigor and reproducibility.

In this review, the term ‘low-cost’ is used in a relative rather than absolute sense, as the cost threshold varies considerably depending on the measurement principle and the reference instrumentation being replaced. Accordingly, studies were selected based on the authors’ explicit framing of their device as low-cost relative to conventional analytical instrumentation, rather than on a fixed price threshold.

A total of 40 studies were analysed to assess the extent to which key validation parameters are addressed, including limits of detection and quantification, calibration strategies, accuracy, precision, repeatability, and comparison with reference methods. In addition, the adoption of open-source practices was evaluated as a proxy for reproducibility and scalability. Based on this analysis, we identified major methodological gaps and inconsistencies in current validation approaches. To address these limitations, we propose a structured validation and reporting framework for low-cost optical sensors, based on improving the reliability, comparability, and reproducibility of measurements. This framework should support the development of more robust sensing systems and facilitate their effective use in distributed and community-based monitoring applications.

## 2. Materials and Methods

**Figure 1:**
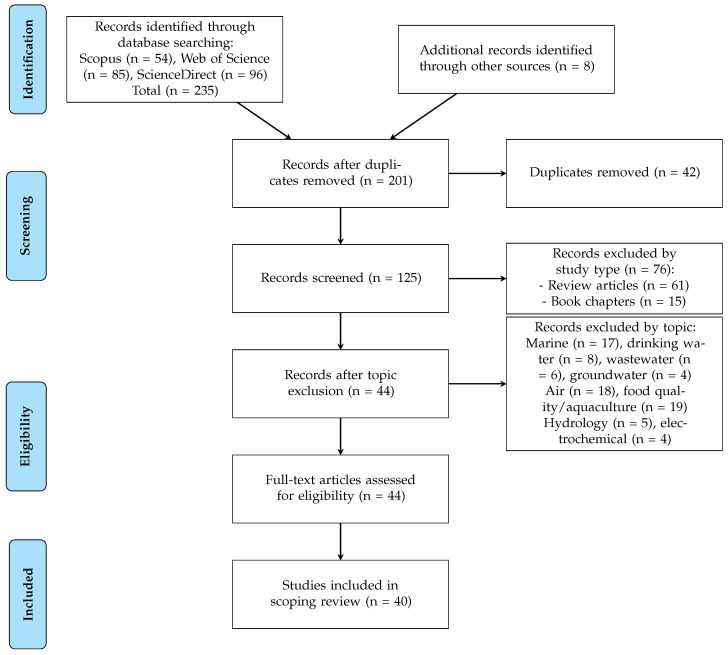
PRISMA-ScR flow diagram of study selection. From 243 initial records (235 identified through database searching and 8 from additional sources), 42 duplicates were removed, leaving 201 records for screening. After excluding 76 records by study type (review articles and book chapters) and 81 records by topic (marine, drinking water, wastewater, groundwater, air, food quality/aquaculture, hydrology, and electrochemical), 44 full-text articles were assessed for eligibility. Following full-text review, 4 records were excluded (non optical low-cost devices or studies without sensor development), resulting in 40 studies included in the scoping review.

The literature search and screening process was conducted in February 2026 following the workflow schematized in Figure 1. No a priori protocol was registered for this review: the study was conducted following the PRISMA-ScR extension for Scoping Reviews [13]. A completed PRISMA-ScR checklist is reported in the Supplementary Materials.

Scopus, Web of Science, and ScienceDirect were used to identify studies focused on the development of low-cost optical sensors for freshwater quality monitoring and potentially deployable within community-based projects. The main research question for this review was: what and how analytical validation parameters are reported, and how these practices align with established guidelines.

The search query was defined as follows: (sensor OR fluorometer OR spectrophotometer OR optical OR turbidity) AND (citizen science OR community-based monitoring) AND (water) AND (low-cost). These keywords were searched within article titles, abstracts, and keywords. In order to expand the search, additional relevant articles were manually searched using the following keywords: in situ monitoring, surface water and miniaturized.

Before the first screening, review articles, book chapters, and non-English language studies were excluded. Only studies published from 2017 were considered in order to focus on recent technological developments and avoid redundancy with earlier systematic reviews addressing foundational concepts. A first literature screening was performed using ASReview (version 2.2), an open-source machine learning-assisted screening tool based on active learning, developed at Utrecht University (Department of Methodology and Statistics) by [14]. After duplicate removal, an initial training set was manually labeled to initialize the model. The algorithm iteratively ranked the remaining records according to their predicted relevance, while all inclusion and exclusion decisions were independently made by two authors. Screening was continued until a predefined stopping criterion was met, corresponding to the point at which no additional relevant studies were identified among consecutively screened records. The use of ASReview aimed to increase screening efficiency while maintaining human control over study selection and minimizing the risk of missing relevant literature. Additional records were excluded if they focused on the following topics: (a) marine waters, (b) groundwater, (c) drinking water, (d) air quality, (e) food quality, (f) aquaculture, (g) hydrology, (h) electrochemical sensing, or (i) wastewater. These eligibility criteria were applied to focus on optical sensors for freshwater quality monitoring designed for community-based projects. Data from resulting studies were classified and extracted by two authors to assess whether they achieved or reported the following characteristics: portability, open-source policy, field testing, validation, comparison with professional instruments or reference methods, and data sharing or connectivity. Studies that did not meet the portability criterion were excluded, as they were considered prototypes or not suitable for field deployment. Finally, the reviewed studies were classified according to their validation approaches, focusing on limits of detection and quantification (LOD and LOQ), accuracy, precision, calibration, repeatability, and statistical comparison with reference methods.

## 3. Overview of Validation and Reporting Practices in Low-Cost Optical Sensors

**Figure 2:**
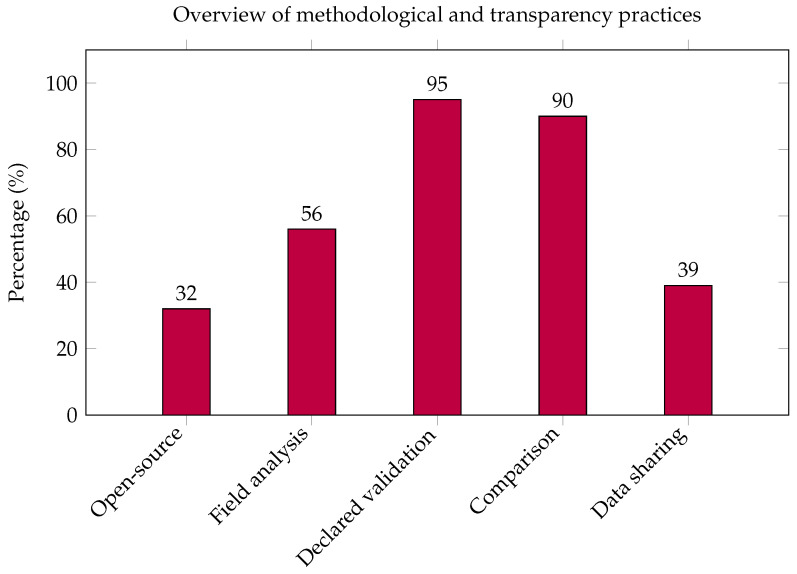
Proportion of studies reporting methodological and transparency practices.

A total of 243 documents resulted from the query (Science Direct = 96, Scopus = 54, Web of Science = 85) and 8 additional records were manually identified through other sources. Duplicates (n = 42) were identified and removed, leaving 201 records for screening. After excluding 76 records by study type (review articles and book chapters) and 81 records by topic, 44 full-text articles were assessed for eligibility. Following full-text review, 4 records were excluded, resulting in 40 studies retained for the final analysis. Each study was evaluated to determine whether it addressed the predefined features listed in Table 1, allowing identification of the most and least considered aspects within the analysed literature (Figure 2). The analytes and measurement principles of the selected studies are summarised in Table 2. The majority of studies reported a validation process for the developed sensors (95%), including comparison with professional instruments or reference methods (90%). Field testing was reported in 56% of studies. In contrast, only 32% of studies provided sufficient information to reproduce the sensors, including 3D-CAD files and software, while 39% included a customized smartphone app or other mechanisms for data sharing, such as Wi-Fi transmission or online platforms for data visualization and analysis.

When deployed in participatory or distributed monitoring frameworks, validation of low-cost sensors becomes a critical step to ensure that analytical performance is consistent with the intended application and measurement context. All analysed articles were systematically compared against a set of key evaluation parameters (Table 3). The results of this evaluation were summarized using a visually coded label system based on three colours. The selection of validation parameters and the colour-coding scheme was guided by recognized standards, including ISO 5725, and the Eurachem validation guidelines, to ensure a consistent and transparent framework for comparison across studies [54,55]. This visualization enables rapid identification of performance metrics that are commonly reported and compliant with standard practices, as well as those that are less frequently assessed in the literature.

### 3.1. Limitations of Common Performance Metrics

The great majority of studies report detection and quantification limits typically calculated either using the calibration curve approach (slope-based method) or by multiplying the standard deviation of repeated blank measurements (usually over ten replicates) by factors of 3 and 10 for LOD and LOQ, respectively. Both approaches are consistent with established validation guidelines. However, while these metrics highlight the sensitivity of newly developed sensors, their standalone reporting may be of limited significance, particularly when they merely reflect multiples of background noise and are not complemented by additional validation metrics such as accuracy, precision, or method comparison [57]. Alternatives to LOD and LOQ such as EC10- or EC50-based approaches are also used in biochemical and biosensing applications depending on the sensing mechanism and response model [58]. However, the analysed literature on optical low-cost sensing systems for water quality monitoring predominantly relied on conventional calibration-based metrics such as LOD, LOQ, and linear regression parameters.

Beyond the widespread reporting of LOD and LOQ, there is another key parameter commonly assessed: the determination coefficient ($R^{2}$), based on the residuals between the predicted and measured concentrations. Research articles on the development of low-cost sensors commonly include the preparation of calibration curves to demonstrate the relationship between the concentration of an analyte and the response of proposed sensors. Showing calibration curves with high $R^{2}$ values is often an accepted standard for demonstrating good sensor performance. However, experiments are often limited to a single calibration curve per analyte, frequently obtained under a single set of experimental conditions. While such an approach provides an experimental proof of concept, it neglects the assessment of repeatability and setup stability over time, which are particularly critical for low-cost sensors intended for repeated or prolonged deployment in the field. Intraday and interday experiments are rarely reported, although recommended by both established validation guidelines and the sensor research community [57]. Moreover, under a linear calibration model (the most frequent in the analysed literature), $R^{2}$ provides a measure of how well the response–concentration data follow a linear fit within the chosen range, and high $R^{2}$ values are routinely reported as proof of excellent sensor capabilities. However, substantial fluctuations are often observed at low concentrations. Because $R^{2}$ is based on squared absolute residuals, observations at higher concentration values typically contribute more strongly to the residual sum of squares. Consequently, large relative errors at low concentrations may correspond to small absolute residuals and thus have limited influence on $R^{2}$. As a result, high $R^{2}$ values may still be obtained even when calibration performance is poor at the low end of the concentration range [59,60]. For these reasons, relying solely on a single $R^{2}$ value as a standard of performance is not sufficient to assess the true reliability of a sensor across its intended concentration range. In contrast, established validation guidelines recommend that calibration performance be evaluated through multiple independent experiments conducted under both repeatability and reproducibility conditions, including intraday and interday measurements across several concentration levels [54,61]. Such approaches enable a more comprehensive assessment of variability, stability, and reproducibility. However, this practice was rarely observed in the analysed literature, where single-condition calibration experiments remained the norm. In some cases, more complex fitting functions were employed without reporting the model equation or explaining the physicochemical rationale for its selection [51]. The choice of calibration model is not arbitrary: an inappropriate fit can systematically distort the derived analytical parameter. For instance, applying a linear model to a response that is inherently nonlinear across the working range will introduce systematic errors in concentration estimates, particularly at the extremes of the range. Established guidelines recommend that the chosen model be explicitly stated and that the selection be justified based on the expected response behaviour of the analytical system [54,61].

### 3.2. Challenges in Accuracy and Precision Assessment

A clear lack of harmonization was observed in the evaluation of accuracy and precision across the studies. Some works conducted structured experiments using multiple solutions at known concentrations, performing repeated measurements (e.g., ten replicates) and reporting statistical indicators such as bias, Relative Standard Deviation (RSD%), Relative Standard Error (RSE%), or recovery [21,40,47]. In contrast, other studies relied exclusively on single-point spike experiments or calculated performance metrics based on a limited number of replicates, sometimes as few as three [31,42,48]. While European guidelines (Eurachem) recommend measuring 10 replicates for accuracy and between 6 and 15 replicates for precision, other guidelines do not specify an exact number [54]. The ICH Q2 validation guideline suggests that accuracy and precision “should be assessed using a minimum of 9 determinations over at least 3 concentration levels covering the specified range” [61]. These guidelines thus define a minimum standard against which the analysed literature can be assessed. The observed heterogeneity in replication levels and concentration coverage suggests that accuracy and precision evaluation in the reviewed studies frequently fell short of the criteria specified by established guidelines, limiting the comparability and reliability of the reported performance metrics.

### 3.3. Measurement Uncertainty and Error Propagation

The correct reporting of uncertainty and error propagation represents a fundamental aspect of analytical method validation, as it provides information on the reliability and comparability of reported results. However, these aspects are generally neglected in the analysed literature, where uncertainty is typically represented through the standard deviation of replicate measurements. While this approach is useful for describing repeatability and short-term precision, it does not provide a complete description of measurement uncertainty, as it neglects additional sources of variability arising from sample preparation, calibration procedures, instrumental effects, and operator-dependent handling.

In particular, key contributors to uncertainty in experimental workflows, such as volumetric operations (such as dilution steps), weighing accuracy, and environmental or operator variability, are rarely reported or quantified. As a consequence, the full uncertainty budget cannot be reconstructed from the available information, limiting the possibility of performing rigorous uncertainty propagation.

More rigorous approaches to uncertainty estimation are well established in analytical chemistry and metrology. These include the calculation of combined standard uncertainty and expanded uncertainty through structured uncertainty budgets. Internationally accepted guidelines, such as the Guide to the Expression of Uncertainty in Measurement (GUM) and the EURACHEM/CITAC guide on quantifying uncertainty in analytical measurement, provide a formal framework for identifying, quantifying, and combining all relevant sources of uncertainty [62,63]. These guidelines highlight that only by systematically accounting for all uncertainty contributions is it possible to obtain a traceable and comparable measurement result. Despite their availability, such methodologies are rarely applied in low-cost and citizen-oriented sensing studies.

## 4. Proposed Framework for Validation of Low-Cost Optical Sensors

While previous studies have emphasized the importance of comparing low-cost sensors with reference instruments, substantial variability remains in how such comparisons are conducted and reported. Some studies omit method comparison entirely, whereas others rely on simple linear regression without further statistical evaluation. More comprehensive approaches, including regression analysis with confidence intervals or relative percent difference (RPD%) calculations across the working range, are less consistently applied. Furthermore, numerical results including $R^{2}$, calibration parameters, and LOD/LOQ values should be reported with a number of digits consistent with the measurement uncertainty, avoiding spurious precision that is not supported by the underlying data Despite the availability of general analytical guidelines, no standardized framework currently exists for the systematic validation and comparison of low-cost optical sensors against established reference methods. To address this gap, we propose a structured set of validation and reporting criteria specifically designed for portable optical sensing systems.

The framework integrates established analytical validation principles with the practical constraints of low-cost sensor development, providing a consistent and reproducible pathway for performance evaluation (Table 4). The proposed framework is based on four core components:

- Calibration and sensitivity assessment, including multi-point calibration across the working range and evaluation of LOD and LOQ;
- Repeatability and reproducibility, assessed through intra-day and inter-day experiments using independent calibration series;
- Accuracy and precision, evaluated across multiple concentration levels using appropriate statistical metrics (RSD%, RSE%, bias, recovery);
- Comparison with reference methods, incorporating regression analysis and RPD% evaluation across the full working range;
- Field testing, validating across potential operational conditions and reporting comparable metrics.

Together, these components provide a comprehensive evaluation of analytical performance, ensuring that sensor outputs are reliable, reproducible, and comparable across studies. Importantly, the framework emphasizes the need for multiple independent experiments and standardized reporting, addressing key limitations identified in the reviewed literature. The adoption of such a structured validation framework enables more transparent interpretation of sensor performance and facilitates comparison between different devices and studies. More broadly, it supports the transition of low-cost optical sensors from proof-of-concept systems to robust analytical tools suitable for real-world deployment.

The proposed framework does not impose significant additional costs beyond standard laboratory practice, since comparison with benchtop instrumentation is already a routine requirement in sensor development, and the remaining steps require only additional measurements using standard materials and time, both of which are inherent to any rigorous analytical study.

To illustrate the practical application of the proposed framework, a representative study from the reviewed literature was assessed against the validation framework presented in this work. Zheng et al. [28] developed a low-cost smartphone-based sensor for the in situ colorimetric detection of nitrite and ammonium in freshwater, specifically designed for citizen science applications. Overall, this study emerges among other studies as one of the most thoroughly validated, yet a few gaps remain when assessed against the proposed framework. Sensitivity was evaluated through the LOD, calculated from 11 blank measurements using the 3σ criterion, which is consistent with established guidelines. However, the LOQ was not reported, representing a minor omission. Regarding calibration, the authors did not explicitly distinguish between intraday and interday experiments; nevertheless, 9 independent calibration curves were constructed across different smartphones, effectively demonstrating the repeatability and cross-device deployability of the method, a particularly relevant feature in a citizen science context where instrument standardization cannot be assumed. Precision was assessed at 2 concentration levels per analyte, with RSD% calculated from more than 7 replicates and extended to include inter-operator variability across 5 users with different levels of expertise, which is especially meaningful for tools intended for non-professional users. Accuracy was evaluated through recovery experiments across ten samples per analyte at two concentration levels, with bias, recovery, and RSD% all reported. Both precision and accuracy were therefore well characterized. Adding a third concentration level would make this aspect fully compliant with the framework proposed herein. The comparison with a benchtop spectrophotometer was conducted on more than 30 samples and supported by regression analysis and a paired t-test, representing a robust approach. Field testing was also reported, though the practical deployability of the sensor within citizen science networks would be further enhanced by sharing open-source CAD files and software, lowering the barrier for independent replication by non-specialist communities.

This example reinforces a key message of this review: achieving rigorous analytical validation does not necessarily require complex instrumentation or elaborate experimental designs. What it does require is collecting a sufficient number of replicates and concentrations rapresentative of the working range and the right analytical parameters. When this is done systematically, even a low-cost, smartphone-based tool intended for non-professional users can meet the standards expected of a validated analytical method.

### 4.1. Technology Readiness and Validation Maturity

The proposed development and validation framework of novel low-cost analytical instruments should be contextualised within the Technology Readiness Levels (TRLs). The TRL scale (Table 5) was introduced by NASA and adopted by the European Commission in 2014 in the context of the Horizon Europe [64]. This scale describes the maturity of a technology along a scale from 1 to 9, from basic principles (TRL 1) to a system proven in operational conditions (TRL 9). In the context of low-cost optical sensors for water quality monitoring, the majority of the analyzed studies can be positioned between TRL 3 and TRL 5. Reaching higher TRL levels (TRL 6–7) requires not only technological development but also the kind of rigorous analytical validation addressed in this framework. In particular, structured assessment of repeatability, reproducibility, accuracy, and field performance are prerequisites for credibly claiming TRL 5 or above. Integrating TRLs assessment into the reporting of low-cost sensor development would therefore provide a useful and internationally recognized reference for evaluating and communicating sensor maturity, facilitating comparison across studies and supporting the transition toward deployable monitoring tools.

### 4.2. Reproducibility and Open-Source Practices

Reproducibility is a critical requirement for low-cost sensor development, particularly in the context of distributed and community-based monitoring. Access to design files, assembly instructions, and source code is essential to enable independent validation, replication, and deployment of sensing systems [65,66]. Without such transparency, the reproducibility and scalability of low-cost sensors remain inherently limited. Open-source hardware and software, supported by accessible documentation and repositories, play a key role in enabling wider adoption and verification of these technologies [67]. In addition to design files and software repositories, the availability of video documentation illustrating sensor assembly, calibration procedures, and real-world deployment can further enhance reproducibility and accessibility. Such visual resources can significantly facilitate the adoption of low-cost sensing technologies by non-specialist users, including citizen scientists and community groups, by providing practical guidance that may be difficult to convey through written documentation alone. Only 27% of the analysed studies adopted an open-source approach to their intellectual property management. Nevertheless, a number of positive examples were present, with developers reporting both open software, 3D-CAD files and assembly instructions, all freely available online through platforms such as GitHub, the Open Science Framework (OSF), or as supplementary files accompanying the articles [19,22,26,27,31,45].

The limited adoption of open-source practices for the development of low-cost optical sensors likely reflects the fact that many researchers may not be familiar with open-source culture, platforms, and associated licensing, which have historically been more established in software and computer science communities. Nevertheless, open-source practices are increasingly gaining traction across scientific disciplines and are increasingly supported by agencies and policy makers, including the European Commission [68]. While sharing materials upon reasonable request has historically represented an accessible intermediate step for researchers less familiar with open repositories, the authors believe that this approach is increasingly insufficient. In the current research environment, AI tools have substantially lowered the technical barriers to publishing on platforms such as GitHub, Zenodo or OSF, making open-source practices achievable regardless of prior experience. We therefore encourage authors to prioritize open publication as the standard approach.

## 5. Technological Innovation and Future Directions for Low-Cost Sensing

The literature analyzed in this study shows a significant technological advance in the development of low-cost optical sensors, with each contribution representing a meaningful innovation, particularly from a technological point of view. They demonstrate the clear opportunities of adopting a DIY and IoT-oriented methodology, taking advantage of microcontrollers or single-board computers and simple optical components such as photodiodes and phototransistors [36,38,41].

Selectivity has been achieved through creative solutions, including HD-DVD diffraction gratings, long-pass optical filters, or RGB detection using smartphone cameras [17,26,33]. The increasing availability of 3D printing has made portability, an essential requirement for in situ measurements, more achievable, and the integration of IoT architecture and artificial intelligence is a promising strategy for enhanced environmental monitoring and data interpretation [12,69].

With adequate analytical validation and reporting, these cutting-edge solutions could make the information provided by citizen scientists more reliable, as a growing number of national and regional Agencies are beginning to use citizen science data in their reporting and policy planning [70,71,72]. Yet technological progress alone is not sufficient: the transition from proof-of-concept devices to deployable analytical tools requires advances in how performance is evaluated and reported.

The adoption of FAIR (Findable, Accessible, Interoperable, Reusable) data practices and the development of community standards for sensor validation represent important directions [73]. Platforms such as Zenodo, Figshare, and the Open Science Framework already offer practical infrastructure for sharing raw data, calibration files, and assembly documentation at no cost, removing one of the main barriers to independent replication. In the context of freshwater monitoring, reporting of validation parameters and comparison with reference methods would improve the comparability of results between different devices. The coordination of such standards through sensor and citizen science-focused journals could provide a shared reference framework for both developers and end users, supporting the broader integration of low-cost sensor data into environmental monitoring programs and policy decisions [70,71,72].

## 6. Limitations of This Study

This work is the first to map and critically evaluate the state of the art of recent low-cost optical sensors for freshwater quality monitoring, with a specific focus on analytical reliability and potential deployment within community-based monitoring. Nevertheless, this study is not devoid of limitations. The research topic addressed in this review is specific, as several sensors designed for other aquatic environments were necessarily excluded from the analysis. Indeed, during the literature screening process, 81 articles were excluded on the basis of topic, as they concerned sensors deployed in environments such as drinking water or marine water systems. These excluded studies represented 33% of the 243 total records retrieved from the databases. This work intentionally focused on low-cost optical sensing systems developed for freshwater monitoring applications. Studies specifically targeting marine water, groundwater, drinking water, or wastewater environments were excluded in order to maintain methodological consistency across the analyzed literature. Although certain sensing principles may be transferable across different aquatic environments, substantial differences in matrix composition, salinity, target working ranges, and deployment strategies can significantly affect sensor operational performance in freshwater. As a consequence, including these systems within a single comparative framework could reduce the consistency of the analysis.

Lastly, part of the relevant literature may have been missed due to the relatively restrictive database query, which combined terms related to optical sensing, citizen science, and freshwater monitoring.

## 7. Conclusions

Low-cost optical sensors have the potential to support community-based freshwater monitoring and international monitoring programs by enabling distributed and cost-effective data collection. However, this study demonstrates that their analytical method validation remains highly heterogeneous and often incomplete, limiting data reliability and comparability. While most studies report sensitivity metrics such as limits of detection and quantification, key parameters including repeatability, accuracy, and precision are inconsistently assessed and rarely aligned with established analytical guidelines. In particular, insufficient replication, and limited evaluation under reproducibility conditions represent critical methodological gaps. To address these limitations, this work proposes a structured validation framework based on standardized evaluation of analytical performance, including intra- and inter-day repeatability, multi-level accuracy and precision, and systematic comparison with reference methods. Adoption of these practices is essential to ensure metrological traceability and enable meaningful comparison and harmonization across studies. Moreover, transparent reporting are key prerequisites for transitioning low-cost optical sensors from proof-of-concept applications to deployable analytical tools. Strengthening methodological rigor will support their integration into distributed monitoring systems, enhancing data quality, spatial coverage, and the effective use of community-generated data in environmental decision-making.

## Figures and Tables

**Table 1:** Summary of characteristics of the reviewed studies. ✓ = reported; – = not reported.

Study	Open-Source	Field Analysis	Validation	Comparison	Data Sharing
[15]	–	–	✓	✓	✓
[16]	–	–	✓	✓	✓
[17]	–	✓	✓	✓	–
[18]	–	–	✓	✓	✓
[19]	✓	–	✓	✓	✓
[20]	–	✓	✓	–	✓
[21]	–	✓	✓	✓	–
[22]	✓	–	✓	✓	–
[23]	–	–	✓	✓	✓
[24]	–	✓	✓	✓	–
[25]	–	✓	✓	✓	✓
[26]	✓	✓	✓	✓	–
[27]	✓	–	✓	✓	–
[8]	–	–	✓	✓	–
[28]	–	✓	✓	✓	✓
[29]	–	–	✓	✓	–
[30]	✓	✓	✓	✓	✓
[31]	✓	✓	✓	✓	–
[32]	–	✓	✓	✓	✓
[33]	–	✓	✓	✓	–
[34]	✓	–	✓	✓	–
[35]	✓	–	✓	✓	–
[36]	–	✓	✓	✓	–
[37]	–	✓	✓	✓	–
[38]	–	–	✓	✓	–
[39]	–	✓	✓	✓	–
[40]	–	–	✓	✓	–
[41]	–	✓	✓	✓	–
[42]	–	–	✓	✓	✓
[43]	–	✓	✓	✓	✓
[44]	–	✓	✓	✓	–
[45]	✓	–	–	–	✓
[46]	–	–	✓	–	–
[47]	✓	✓	✓	✓	✓
[48]	–	✓	✓	✓	–
[49]	✓	✓	✓	✓	✓
[50]	–	✓	✓	✓	–
[51]	✓	✓	✓	✓	–
[52]	✓	✓	✓	✓	✓
[53]	✓	✓	✓	✓	✓

**Table 2:** Measurement principles and analytes.

Study	Analyte	Measurement Principle
[15]	Dissolved Organic Matter	Fluorescence
[16]	Turbidity	Scattering
[17]	Quinine + Sulfate	Fluorescence + Scattering
[18]	Fluoride	Colorimetry
[19]	Vitamin B12 + Phosphate + $H_{2}O_{2}$	Colorimetry + Spectrophotometry
[20]	Turbidity	Scattering
[21]	1,3-DCP	Colorimetry
[22]	Free Chlorine	Spectrophotometry
[23]	*Cryptosporidium* RNA	Colorimetry
[24]	Phosphate	Colorimetry
[25]	Cr(VI)	Colorimetry
[26]	Chlorophyll	Fluorescence + Spectrophotometry
[27]	Nitrate	Colorimetry
[8]	Cu + Fe	Colorimetry + Spectrophotometry
[28]	Nitrite + Ammonium	Colorimetry
[29]	Chemical Oxygen Demand	Spectrophotometry
[30]	${\mathrm{NO}}_{2}$	Colorimetry
[31]	Nitrite	Fluorescence
[32]	Phosphate + Cr(VI) + Fe + Zn + Turbidity	Colorimetry
[33]	Nitrate + Ammonium + Phosphate	Colorimetry
[34]	Uranine	Fluorescence
[35]	Turbidity	Scattering
[36]	Chlorophyll	Fluorescence
[37]	Sulfide	Colorimetry
[38]	Fe	Colorimetry
[39]	Antibiotics	Fluorescence
[40]	Nitrate	Spectrophotometry
[41]	Chlorophyll	Fluorescence
[42]	Turbidity	Scattering
[43]	Phosphate	Colorimetry
[44]	Fe + Cu + ${\mathrm{PO}}_{4}$ + ${\mathrm{NH}}_{4}$ + ${\mathrm{NO}}_{3}$ + ${\mathrm{NO}}_{2}$ + ${\mathrm{SO}}_{4}$	Colorimetry
[45]	Turbidity	Scattering
[46]	Microplastics	Fluorescence
[47]	Microcystin-LR	Bioluminescence
[48]	Microplastics	Fluorescence
[49]	Phosphate + Nitrate	Colorimetry
[50]	Chlorophyll	Fluorescence
[51]	Turbidity	Fluorescence
[52]	Bilirubin	Fluorescence
[53]	Dissolved Organic Matter	Fluorescence

**Table 3:** Systematic comparison of major studies that develop and validate low cost optical sensors for water quality. Colour coding: ⬤ parameter compliant with established validation guidelines; ⬤ parameter partially reported or not fully compliant; ⬤ parameter not reported.

Study	LOD/LOQ	Accuracy	Precision	Repeatability	Comparison
[15]	⬤	⬤	⬤	⬤	⬤
[16]	⬤	⬤	⬤	⬤	⬤
[17]	⬤	⬤	⬤	⬤	⬤
[18]	⬤	⬤	⬤	⬤	⬤
[19]	⬤	⬤	⬤	⬤	⬤
[20]	⬤	⬤	⬤	⬤	⬤
[21]	⬤	⬤	⬤	⬤	⬤
[22]	⬤	⬤	⬤	⬤	⬤
[23]	⬤	⬤	⬤	⬤	⬤
[24]	⬤	⬤	⬤	⬤	⬤
[25]	⬤	⬤	⬤	⬤	⬤
[26]	⬤	⬤	⬤	⬤	⬤
[27]	⬤	⬤	⬤	⬤	⬤
[28]	⬤	⬤	⬤	⬤	⬤
[29]	⬤	⬤	⬤	⬤	⬤
[31]	⬤	⬤	⬤	⬤	⬤
[32]	⬤	⬤	⬤	⬤	⬤
[33]	⬤	⬤	⬤	⬤	⬤
[8]	⬤	⬤	⬤	⬤	⬤
[34]	⬤	⬤	⬤	⬤	⬤
[35]	⬤	⬤	⬤	⬤	⬤
[56]	⬤	⬤	⬤	⬤	⬤
[36]	⬤	⬤	⬤	⬤	⬤
[37]	⬤	⬤	⬤	⬤	⬤
[38]	⬤	⬤	⬤	⬤	⬤
[39]	⬤	⬤	⬤	⬤	⬤
[40]	⬤	⬤	⬤	⬤	⬤
[42]	⬤	⬤	⬤	⬤	⬤
[41]	⬤	⬤	⬤	⬤	⬤
[43]	⬤	⬤	⬤	⬤	⬤
[44]	⬤	⬤	⬤	⬤	⬤
[45]	⬤	⬤	⬤	⬤	⬤
[46]	⬤	⬤	⬤	⬤	⬤
[47]	⬤	⬤	⬤	⬤	⬤
[48]	⬤	⬤	⬤	⬤	⬤
[49]	⬤	⬤	⬤	⬤	⬤
[50]	⬤	⬤	⬤	⬤	⬤
[51]	⬤	⬤	⬤	⬤	⬤
[52]	⬤	⬤	⬤	⬤	⬤
[53]	⬤	⬤	⬤	⬤	⬤

**Table 4:** The key steps in the proposed validation framework for water quality applications.

Step	Reps	Levels	Parameters	Purpose
LoD & LoQ	≥10	Near detection limits	LoD = 3σ/slope, LoQ = 10σ/slope	Sensitivity
Repeatability	≥4 (intra/inter)	≥5 range	RSD%, RSE%, regression	Precision check
Accuracy & Precision	6–10	≥3 range	Bias, Recovery, RSD%	Performance evaluation
Reference comparison	≥3	≥3 range	Regression, RPD%	Method agreement
Field testing	6–10	≥3 range	Bias, Recovery, RSD%	Real-world validation
Uncertainty & error propagation	6–10	Full working range	reporting using one of the established approaches (GUM/EURACHEM)	Metrological traceability

**Table 5:** Technology Readiness Levels (TRLs) and associated development goals (European Commission framework).

TRL	Specific Goal (European Union)
1	Basic principles observed
2	Technology concept formulated
3	Experimental proof of concept
4	Technology validated in laboratory
5	Technology validated in relevant environment
6	Technology demonstrated in relevant environment
7	System prototype demonstration in operational environment
8	System complete and qualified
9	Actual system proven in operational environment

## Data Availability

No new data were created or analyzed in this study. Data sharing is not applicable to this article.

## Supplementary Materials

The following supporting information can be downloaded at: https://www.mdpi.com/article/10.3390/s26123846/s1, Table S1: Preferred Reporting Items for Systematic reviews and Meta-Analyses extension for Scoping Reviews (PRISMA-ScR) Checklist.
